# Skin lesion classification system using a K-nearest neighbor algorithm

**DOI:** 10.1186/s42492-022-00103-6

**Published:** 2022-03-01

**Authors:** Mustafa Qays Hatem

**Affiliations:** Renewable Energy Department, Technical Institute of Baqubah, Middle Technical University, Dyala, 32001 Iraq

**Keywords:** Machine learning, Skin disease, K-nearest neighbor, Skin detection, MATLAB, Graphical user interface

## Abstract

One of the most critical steps in medical health is the proper diagnosis of the disease. Dermatology is one of the most volatile and challenging fields in terms of diagnosis. Dermatologists often require further testing, review of the patient’s history, and other data to ensure a proper diagnosis. Therefore, finding a method that can guarantee a proper trusted diagnosis quickly is essential. Several approaches have been developed over the years to facilitate the diagnosis based on machine learning. However, the developed systems lack certain properties, such as high accuracy. This study proposes a system developed in MATLAB that can identify skin lesions and classify them as normal or benign. The classification process is effectuated by implementing the K-nearest neighbor (KNN) approach to differentiate between normal skin and malignant skin lesions that imply pathology. KNN is used because it is time efficient and promises highly accurate results. The accuracy of the system reached 98% in classifying skin lesions.

## Introduction

Dermatology is the study of various skin diseases and pathologies, including skin cancer. Diagnosing in this branch of science depends primarily on the physical outer appearance of the skin. For this reason, skin disease diagnosis is based on different types of imaging, such as ultrasound, dermatoscopy, and reflectance confocal microscopy [1]. Challenges other than diagnosis are also present, such as the overwhelming amount of data usually collected from an individual personally and from their imaging results [2, 3].

Different artificial intelligence and machine learning technologies have been used, especially in the past two decades, to advance medicine in general, whether in diagnosis or treatment [4]. Furthermore, they have been applied in many domains, such as power and electricity markets and renewable energy [5–9]. Artificial intelligence includes machines or programs that mimic the human brain in its method of thinking. In contrast, machine learning is a technology that uses several smart algorithms that can be taught to solve problems, process large datasets, and even develop their own selves [2, 10].

Dermatoscopy is one of the most important methods for detecting and classifying skin cancers through imaging. Automated analysis of these resultant images can be performed as a method to help dermatologists make better decisions. The latter is data-based to ensure that an optimal course of action is taken towards the patient. This analysis can be facilitated by new technologies such as convolutional neural networks (CNNs) [11]. CNN technology has reached an “expert level” in the classification of various types of skin cancer as well as other skin pathologies [12].

Other innovative techniques such as linear discriminant analysis, support vector machine (SVM), naive Bayes classifier, K-nearest neighbor (KNN), and deep learning algorithms are also widely used for the classification of skin diseases. When implementing machine learning algorithms, the images are uploaded and processed, and most importantly, the features from the image are selected for analysis. Therefore, the feature selection step is the most significant and vital step [11].

In fact, the entirety of the healthcare system is being advanced using rising technologies such as deep neural networks and machine learning algorithms [13]. The importance of these new technologies lies in their ability to recognize patterns, which is essential in the medical field. For example, neural networks, such as the multilayer perceptron (MLP)-artificial neural network (ANN), when combined with the world cup optimization algorithm, are extremely effective in detecting melanoma from images [14]. In addition, some studies used the grey wolf optimization for MLP-ANN to detect melanoma and achieved great efficiency and accuracy [15]. Similarly, a CNN optimized via the satin bowerbird optimization algorithm has shown effectiveness in detecting cancer [16]. Thus, it is evident that computer-based methods and machine learning algorithms are quite beneficial in the medical field owing to the implication of data mining strategies, as well as logical analysis and assisted feature selections [17]. In addition, computers or machines can achieve the same results (if not better) with easy data management and in a short time [18].

For the optimization processes, uncertainty quantification (UQ) is extremely essential and plays a significant role in the decision-making steps. Ensemble machine learning techniques as well as Bayesian approximations depend on UQ, especially in computer vision, image processing, and medical image analysis [19]. Some studies focus on this specific concept to create better models such as a binary residual feature fusion, implemented with Monte Carlo dropout used for medical image classification [20]. An uncertainty-aware model has also been proposed and successfully executed for the detection of cancer in breast histology images [21].

In this study, to ensure accurate and easy diagnosis of skin lesions, the proposed system implements the KNN as the classification method for differentiating between normal skin and malignant skin lesions that imply pathology. KNN is used because it is time efficient when it comes to generating results. Moreover, KNN is an algorithm that provides results that are easy to interpret and versatile and can be used for classification and regression. Among other machine learning models, KNN is a very accurate algorithm.

### Related works

Some research papers for automatic benign and malignant skin lesion detection are presented in this section.

Kassem et al. [22] discussed the classification of skin lesions into eight classes using a deep CNN and transfer learning. The eight classes were dermatofibroma, melanocytic nevus, vascular lesions, basal cell carcinoma, actinic keratosis, benign keratosis, squamous cell carcinoma, and melanoma. The system proposed in the study achieved high levels of accuracy (94.9%), precision (80.3%), specificity (97.0%), and sensitivity (79.8%).

Balaji et al. [23] analyzed the performance of several neural network architectures that were used for skin cancer classification via a firefly optimization strategy. The system involved fuzzy c-means for segmentation, followed by firefly optimization for feature selection. The proposed model was evaluated in terms of specificity, sensitivity, accuracy, and satisfactory results were obtained.

Amin et al. [24] proposed an integrated design for localizing and classifying skin cancers. Initially, images were preprocessed and resized, followed by bio-orthogonal 2D wavelet transformation, where Otsu’s method was used for segmenting the images. Subsequently, the features were extracted using pre-trained AlexNet and VGG-16. The results of the study showed that it outperformed the other methods in terms of accuracy.

Ali et al. [25] introduced a deep CNN model to accurately classify benign and malignant skin lesions. They compared their model with other transfer learning models used. The proposed method was faster, more reliable, and more robust with 93.16% training and 91.93% testing accuracy.

Thomas et al. [26] were the first to use interpretable deep learning systems simultaneously for multi-class segmentation and classification of non-melanoma skin cancer. They classified the tissue for characterization into 12 dermatological classes. These classes included hair follicles, sweat glands, and well-defined stratified skin layers. High accuracy was obtained (˃ 93%) when the entire tissue was classified. In this way, the method used was able to perform routine pathologist tasks, such as surgical margin clearance assessments.

Dai et al. [27] reported using mobile device technologies for private classification of new data. The classification process was performed using an Inference App, which was stored on the device and specifically pre-trained for classification. The paper described the basic concepts of the project by conducting a case study, which also assessed the efficiency of the system, especially focusing on skin cancer, which is one of the most widely spread human malignancies.

Kumar et al. [28] suggested a technique for evaluating the presence or absence of melanoma in a tested sample. The study demonstrated the following steps: the preprocessed images were used to collect labeled data before they were flattened and subjected to pixel extraction. The extracted pixel intensities could then be gathered in an array and stored in a specific database. An SVM with labeled data used a convenient kernel to successfully classify the samples by relying on previously trained data. The proposed system showed a 90% accuracy level of classification.

Using a pre-trained deep CNN, El Saleh et al. [29] introduced an automated facial skin disease identification system. Their system was able to achieve an 88% accuracy rate in identifying eight different facial skin diseases. They used specific image preprocessing techniques to redevelop the images. The images were then collected from various sources and resized to increase the size of the database. The preprocessed, extensively modified images were then used as sets for training and further validation.

Patnaik et al. [30] presented a new strategy that employed deep learning technologies, such as computer vision procedures, to predict numerous forms of skin diseases automatically. To determine the skin disease type, the system framework depended on the voting results of three different publicly accessible image recognition designs: InceptionV3, Inception ResnetV2, and MobileNet. The accuracy level of this system was considered high because the implemented models underwent extensive training to identify up to 1000 different groups. This particular method, like most others, applied feature extraction as well as the training and testing phases.

Rathod et al. [31] proposed an automated image-based framework governed by machine learning classification to recognize different skin pathologies. The framework depended on computational techniques to examine, process, and transfer image data based on different image features. The skin images were filtered and processed to eliminate excess noise and enhance the image. Innovative techniques, such as CNNs, were used for feature extraction. To classify the images, the softmax classifier algorithm was used. The system was fast and highly precise compared to other conventional approaches that are used for detecting dermatological diseases.

Kumar et al. [32] used a two-step approach incorporating computer vision in addition to machine learning to detect clinical histopathology cases. Initially, the image of the skin disease was preprocessed and exposed to feature extraction using multiple techniques. Subsequently, machine learning algorithms were applied to classify diseases based on the data. Table 1 summarizes the different image classification methods.

**Table 1:** Different image classification methods

Applied method or technique	Accuracy of measurements (%)	Remarks	Reference
CNN	90.0	Skin diseases can be diagnosed and classified using the same CNN technique	[31]
InceptionV2, InceptionV3, MobileNet	88.0	Recommended for mobiles and embedded applications as MobileNet is light weight architecture and fast model	[30]
CNN, VGG-16 model	88.0	The accuracy of the system can be improved by increasing the size of dataset and new deep neural network models can also be considered	[29]
Image processing, SVM	90.0	The system can be extended for classifying other diseases	[28]
CNN using TensorFlow	75.2	The system can be implemented in android device using Tensorflow lite	[27]
Deep CNN in addition to GoogleNet	94.9	The model are able to detect images that do not belong to the eight used classes (classified as unknown images)	[22]
Neural and fuzzy approach	94.5	The proposed method improves the performance by 4.9%	[23]
Otsu algorithm, Alex and VGG-16 model	99.0	Better results were achieved compared to existing methods	[24]
Deep CNN	91.9	The used model is more reliable and robust compared with existing transfer learning models	[25]
CNN, Random Forest, KNN, Single-layered perceptron	93.6-97.9	The proposed method can perform several routine pathologist tasks	[26]

### System overview

**Fig. 1:**
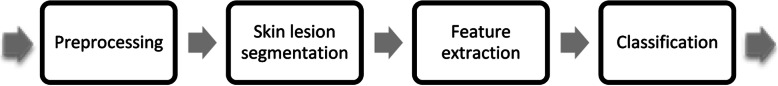
Phases of the detection system

**Fig. 2:**
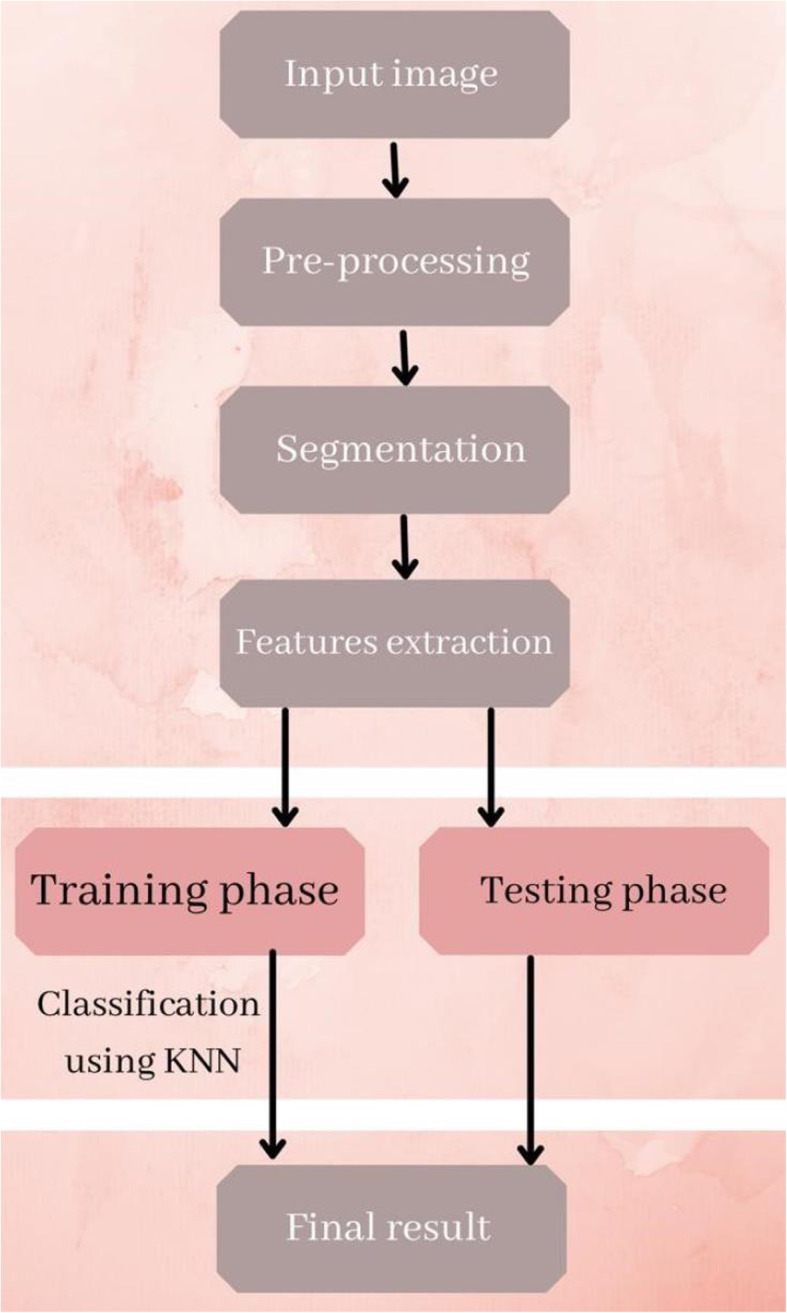
System architecture for the proposed work system

The system comprises four phases, as shown in Fig. 1. The following enhancement and morphological operations were applied to the test images. Using morphological operations, we eliminated noise, sharpened the details of the infected area, and changed the contrast of the image, thus making it easier to identify key features. Adaptive thresholding was used in the segmentation. Mean (fast Fourier transform), standard deviation, histogram-based mean and standard deviation, edge-based pixel count of area and hole, and edge-based logarithmic pixel count of area and hole were all used in feature extraction. This is a key attribute that distinguishes diseases from one to another. The KNN classifier was used to predict the malignancy of a skin lesion using statistical texture features. Skin images in the test set were categorized by comparing them to the skin images in the training set [33]. The training collection included images of normal and cancerous skin, as well as images of skin diseases. The local features obtained in the previous step were used for comparison. Compared with conventional classifiers, such as decision trees and neural networks, KNN has many advantages [34]. The modules involved in the proposed work are described in Fig. 2 and in the following section.

## Methods

The process of skin classification involved the following steps.

### Image acquisition

The proposed system used dermoscopic images as the input. Dermatoscope is an instrument that takes pictures of skin lesions (body parts) and makes it much easier to diagnose skin diseases. A skin disease database can also be created so that it can be easily accessed online [35].

### Preprocessing

First, the skin file was preprocessed. Skin images usually have pathological noise and a variety of texture backgrounds, making extraction impossible. Preprocessing the images involved two basic steps: hair removal and image enhancement.

The goal of this step was to improve and enrich the image quality to increase the certainty and make the segmentation of the skin lesion much easier. As a result, the morphological ‘closing’ operation was used to achieve the purpose of filtering out the shape and structure of the image.

### Segmentation

The primary goal of medical image segmentation is to distinguish tumors from context. The skin lesion was segmented using the thresholding method, which involved setting all pixels with intensity values greater than the threshold to a foreground value. The background values were applied to the remaining pixels. Traditional thresholding strategies apply a single global threshold to all the pixels. In contrast, adaptive thresholding adjusts the threshold value dynamically as the picture progresses. In this specific step, adaptive thresholding was used to remove the skin image.

### Feature extraction

Feature extraction is frequently required for effective segmentation. Lesions are of various sizes and properties. Therefore, features must be extracted before they can be fed into the classification level [36]. A vector of features converted the skin mole to intensity values, and the number of extracted properties determined its dimensions. To generate these features, many statistical properties were computed. The segmented dermoscopic lesions were then used to extract the features. To determine the parameters, mathematical terms, such as the mean value, were used.

### Classification

Skin lesions were recognized and classified according to a set of characteristics. A broad variety of classifiers has been investigated over the year. However, the KNN classifiers proved to have superior performance; hence, they were chosen for use in this system for skin lesion classification.

To further illustrate, the KNN has a shorter execution time and a higher accuracy than other widely used methods, such as the hidden Markov model and kernel process, as well as SVM classifiers [37].

### Proposed system

Nearest neighbor classification is one of the easiest classification techniques in the image space. Thus, when the test image is considered, it is assigned a label of the closest point of the learning set according to the spaces in the image. By default, in KNN, the Euclidean distance measurement is often used to calculate the distance between several data points within an image, and each pixel is assigned a distance. The Euclidean distance between two pixels is referred to as the ‘distance’ [38].

Following the extraction process, the extracted features are added directly to classifiers or machine learning tools for distribution into two classes. The entire procedure consists of two stages or phases. The first is for training, and the second is for testing the images against the training dataset [39].

Training phase: the patterns of benign and malignant images in terms of features and class labels were fed to the classifiers for training. The feature attributes that were previously extracted were used to train the sample images of approximately 40 melanoma (type = 1) and 40 normal (type = 0) skin images. These data points were then plotted in the feature space [40].

Testing phase: an unknown test pattern was fed and was later classified and plotted in the feature space again using the information acquired during the training process. A feature space is an abstract space in which each sample image is depicted as a point in an n-dimensional space with its attributes. The number of features used to characterize the patterns determines its size. The total number of images in the training database was 40.

The optimal K value was determined using the accuracy plot method. Moreover, in this study, the k-means algorithm had a time complexity of O(*n*^2^), where *n* is the input data size.

## Results and Discussion

Graphical user interface (GUI) is a relatively simple software, which is easily manageable and offers features such as “point and click support” to the user. The GUI allows the systems or applications to run by simulation without the need of the user to constantly learn, type, or run the code whenever they wish to use the application.

**Fig. 3:**
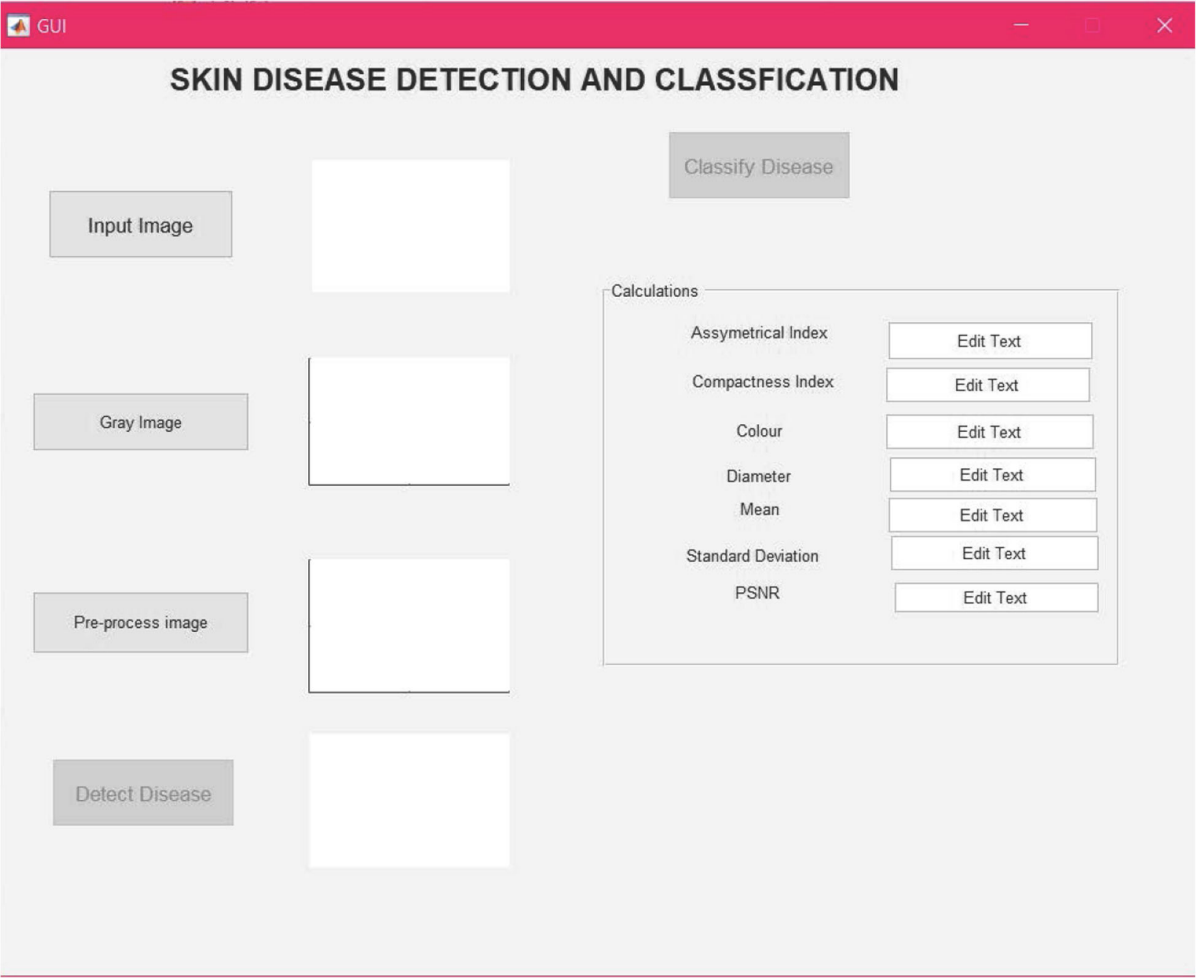
GUI for the proposed method

In this project, the MATLAB GUI was chosen as the method to acquire and upload different images, extract their features, and perform segmentation with ease and time efficiency, as shown in Fig. 3.

From Fig. 3, it is clear that the GUI contains many different buttons for different purposes, such as an input image, gray image, preprocessing image, detection of disease, and classification of disease buttons. Hence, for GUI users, direct image uploading into the application can be further used by the system processes. For this reason, the results of each step can be shown on the GUI screen itself as images or displayed in the relevant message box.

**Fig. 4:**
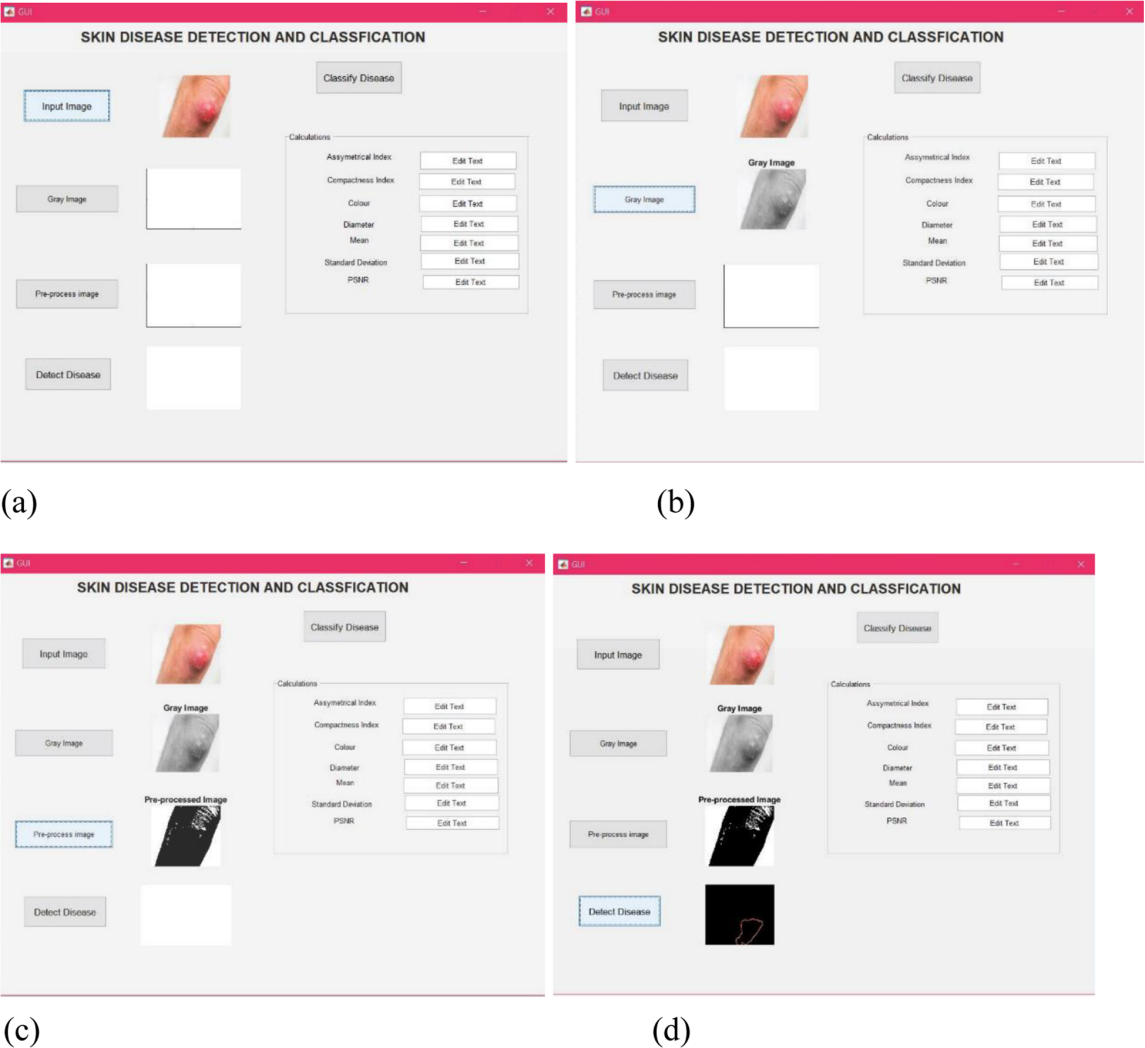
The results of each step on the GUI screen. **a**: Result for input image; (**b**): Result for gray image button; (**c**): Result for pre-processing image button; (**d**): Results of the detection of disease button.

Moreover, the first step is to use the input image button to capture an image of the affected skin region. Then, it can be subjected to preprocessing [Fig. 4(a)]. The second step is to activate the gray image button. Grey image contrast enhancement is performed to improve the clarity of the image, followed by red/green/blue (RGB) to grayscale conversion [Fig. 4(b)]. The third step is to employ the preprocessing image button, which initializes the performance of two smaller processes. Contrast enhancement is performed using histogram equalization and grayscale conversion. In this process, a raw binary image is transformed into an RGB matrix form to distinguish each pixel from its neighbor. Then, the RGB matrix is converted into a grayscale matrix, as shown in Fig. 4(c). The fourth step is to detect the disease [Fig. 4(d)]. This step involves feature extraction, after which the most convenient details in the image are considered, and other details are discarded. This process is performed to make the detection or classification step much easier.

**Fig. 5:**
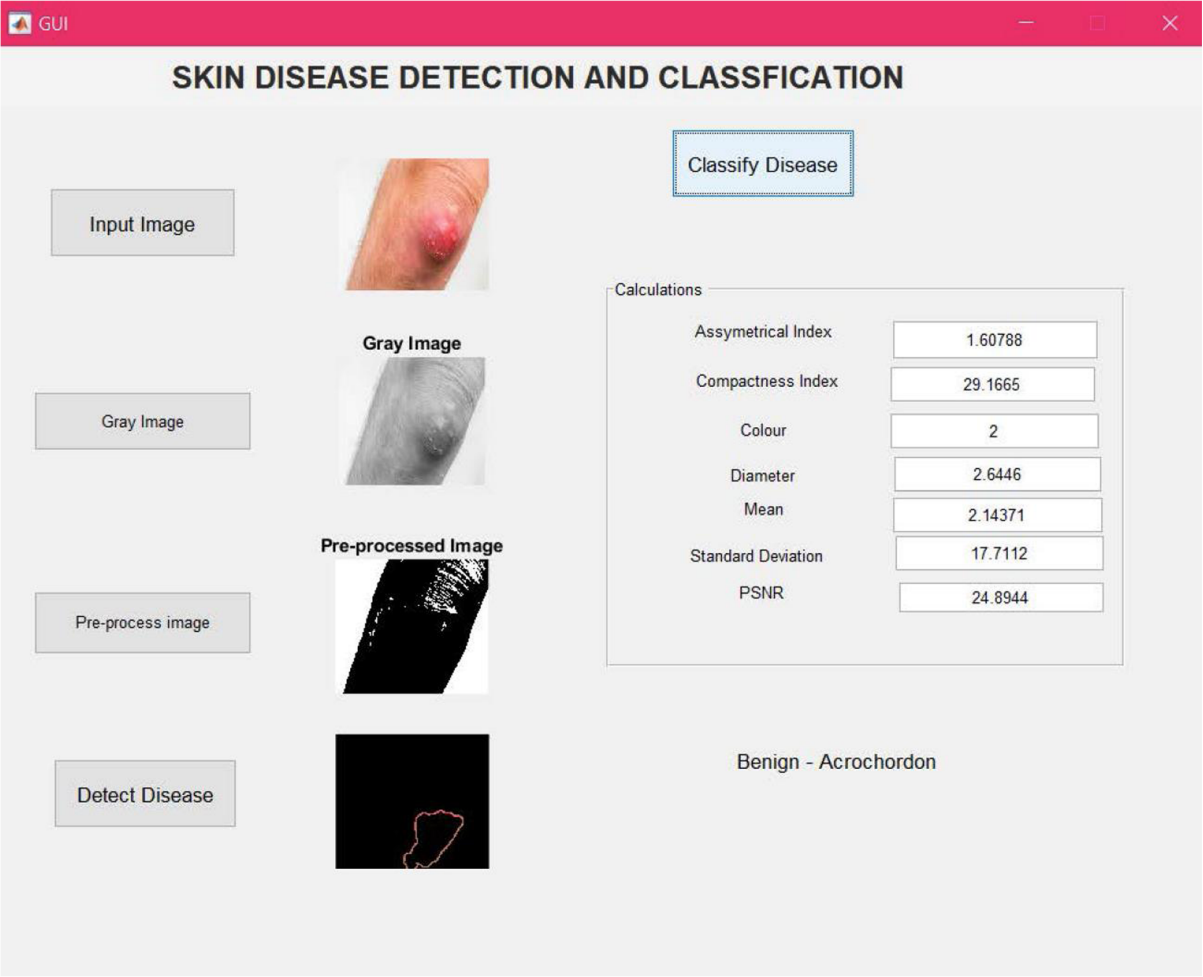
Result for classify disease button

Finally, after all these processes were successfully executed, classification of the disease button is enabled. By pressing this button (Fig. 5), the output results show various calculation results, such as the asymmetrical index, mean value, compactness index, color, diameter, standard deviation value, and peak signal-to-noise ratio. These calculations are further used to classify the type of skin disease present in the input image.

Using the proposed system, we achieved 98% accuracy. Comparing the results with those of previous studies (Table 1), the new system has very high accuracy, which is comparable to the accuracy of the best method. Furthermore, the advantages of the proposed system are that it is easy to implement and fast (no training period needed) as it is based on the KNN algorithm, and as a result, new data can be added seamlessly without affecting the accuracy. However, the disadvantages are that it does not work well with a large dataset and is sensitive to the noise present in the dataset. The system can be further improved by using ensemble learning methods or evolutionary algorithms that guarantee even higher accuracy and faster results.

## Conclusions

Diagnosis of skin pathologies is a complex procedure that requires time and may lead to misdiagnosis. For this reason, automated systems based on machine learning serve as important tools to assist physicians in properly classifying skin lesions. The proposed system consists of four major phases: image preprocessing, segmentation, feature extraction, and classification. The code of the system is run on MATLAB and is based on the KNN algorithm. The system is presented in a user-friendly GUI that allows step-by-step classification, with visualization of the statistical features that are used for classification. Upon testing, the system achieved 98% accuracy. In the future, the system can be improved by using ensemble learning methods or evolutionary algorithms that guarantee even higher accuracy and faster results.

## Data Availability

The datasets used and/or analyzed during the current study are available from the corresponding author on reasonable request.

## Abbreviations

KNN: K-nearest neighbor
CNN: Convolutional neural network
SVM: Support vector machine
MLP: Multilayer perceptron
ANN: Artificial neural network
UQ: Uncertainty quantification
GUI: Graphical user interface
RGB: Red/green/blue
